# Facile generation of giant unilamellar vesicles using polyacrylamide gels

**DOI:** 10.1038/s41598-020-61655-2

**Published:** 2020-03-16

**Authors:** Eric Parigoris, Daniel L. Dunkelmann, Allan Murphy, Nino Wili, Andres Kaech, Claudia Dumrese, Noemi Jimenez-Rojo, Unai Silvan

**Affiliations:** 1 University Hospital Balgrist, University of Zurich, Zürich, Switzerland; 2 Institute for Biomechanics, ETH Zurich, Zürich, Switzerland; 3 Wallace H. Coulter Department of Biomedical Engineering, Georgia Institute of Technology, Atlanta, USA; 4 MRC Laboratory of Molecular Biology, Cambridge, United Kingdom; 5 Laboratory of Physical Chemistry, ETH Zurich, Zürich, Switzerland; 6 Center for Microscopy and Image Analysis, University of Zurich, Zurich, Switzerland; 7 Flow Cytometry Facility, University of Zurich, Zurich, Switzerland; 8 NCCR Chemical Biology, Department of Biochemistry, University of Geneva, Geneva, Switzerland

**Keywords:** Biological techniques, Biological models, Lipids

## Abstract

Giant unilamellar vesicles (GUVs) are model cell-sized systems that have broad applications including drug delivery, analysis of membrane biophysics, and synthetic reconstitution of cellular machineries. Although numerous methods for the generation of free-floating GUVs have been established over the past few decades, only a fraction have successfully produced uniform vesicle populations both from charged lipids and in buffers of physiological ionic strength. In the method described here, we generate large numbers of free-floating GUVs through the rehydration of lipid films deposited on soft polyacrylamide (PAA) gels. We show that this technique produces high GUV concentrations for a range of lipid types, including charged ones, independently of the ionic strength of the buffer used. We demonstrate that the gentle hydration of PAA gels results in predominantly unilamellar vesicles, which is in contrast to comparable methods analyzed in this work. Unilamellarity is a defining feature of GUVs and the generation of uniform populations is key for many downstream applications. The PAA method is widely applicable and can be easily implemented with commonly utilized laboratory reagents, making it an appealing platform for the study of membrane biophysics.

## Introduction

Giant unilamellar vesicles (GUVs) are widely used in the study of processes involving biological lipid membranes. With diameters resembling the size of eukaryotic cells, GUVs enable myriad applications as simplified model cellular systems. GUVs are used in applications ranging from the analysis of lipid-lipid interactions, where microscopic phase separation of lipids is followed using fluorescent dyes^1,2^, to the reconstitution of cellular machineries^3–5^.

To date, a variety of approaches ranging from simple gentle hydration to elaborate microfluidic techniques have been developed to create these cell-sized vesicles^6–11^. Historically, the generation of vesicles under physiologically relevant conditions, while maintaining the unilamellarity and integrity of the lipid membrane, has been a challenging task. While several more recent studies have successfully developed protocols for the generation of GUVs using buffers of physiological ionic strength^12,13^, these have not been universally adopted.

Electroformation, a method in which the swelling of lipids deposited on electrodes placed within an aqueous solution is modulated using an externally applied electric field, has become one of the most frequently utilized methods for GUV production^14^. Although electroformation has been recently adapted for the generation of GUVs from charged lipid types and using buffers of physiological ionic strength, the protocol requires careful optimization depending on the lipid charge and buffer used, and is generally not recommended for solutions of high ionic strength^12,15–17^. An additional disadvantage of this method when used for the *in vitro* reconstitution of cellular machineries is that the required electric fields might affect biomolecules^18^. To overcome these limitations, Horger and colleagues described a method in 2009 using partially dried hybrid gels of agarose and lipids to generate large populations of free-floating GUVs. The agarose protocol allowed for the use of buffers of physiological ionic strength and was compatible with polar and apolar lipid types^19^. However, the use of agarose hydrogels, which are not covalently linked, results in agarose incorporation inside of the lumen and thus leads to inconsistent mechanical properties^20^. This challenge was later circumvented by exchanging the agarose gels with different covalently linked hydrogels, such as poly(vinyl alcohol)^21^ or dextran(ethylene glycol)^22^. Although the problem of gel incorporation into the formed vesicles was mitigated, these techniques are complex and require chemicals that are uncommon in most laboratories, therefore hindering their broad adoption.

In the present work, we report a gentle hydration method for the generation of large populations of cell-sized free-floating GUVs from hybrid films of dried polyacrylamide (PAA). Interestingly, we demonstrate that the use of PAA gels produces a significantly higher proportion of unilamellar vesicles when compared to similar methods described to date. As with the agarose gel method, gentle hydration of the hybrid PAA-lipid gels also allows for the generation of GUVs using common chemicals present in the majority of laboratories.

## Results

**Figure 1:**
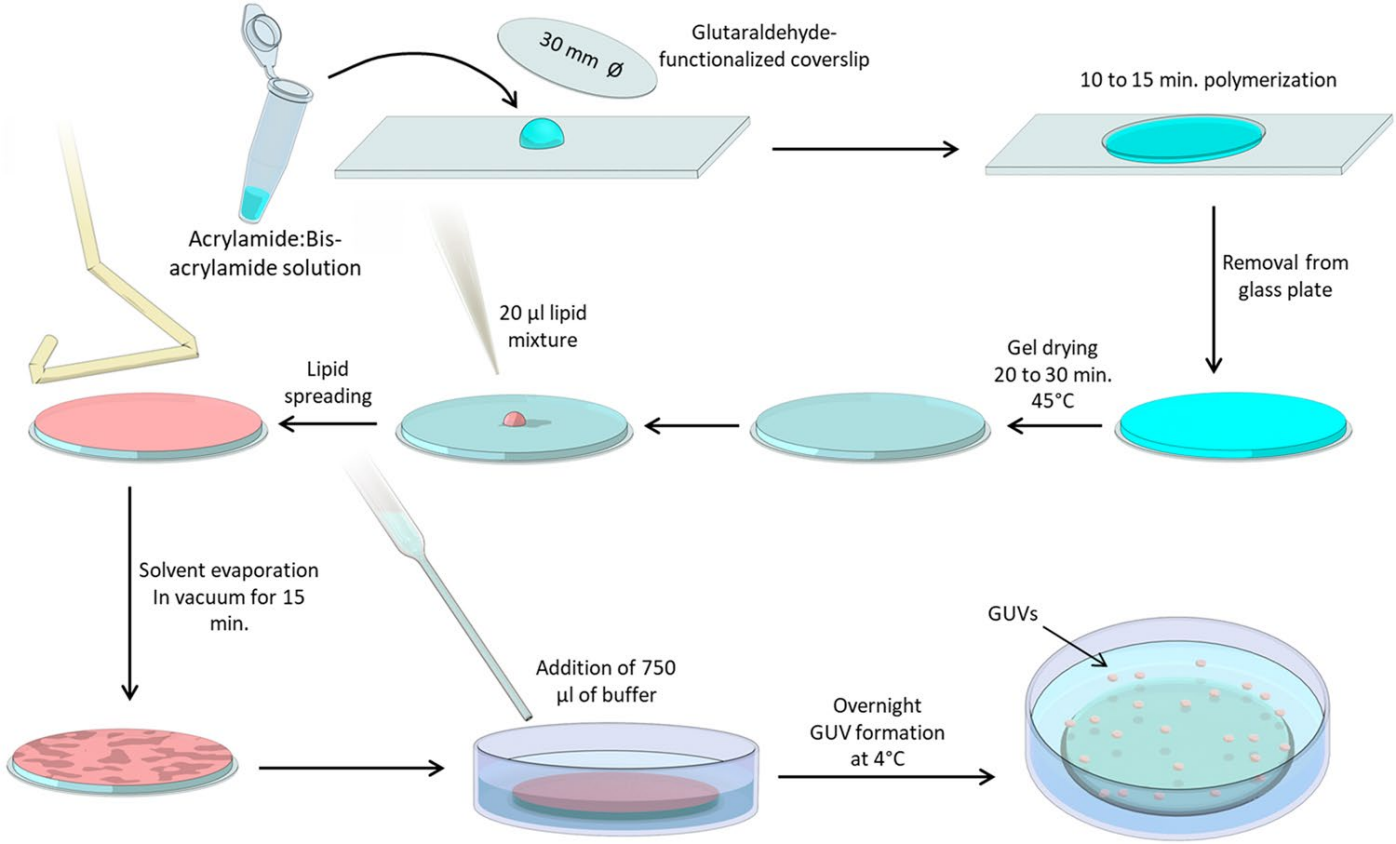
Procedure of forming GUVs using dried PAA gels. A solution containing the desired concentrations of acrylamide and bisacrylamide (Table 1) was prepared and degassed in a vacuum chamber for 30 minutes. 1 µL of TEMED and 10 µL of 10% APS were added per 1 mL of PAA solution, and 45 µL of solution was subsequently transferred onto a clean glass plate. Functionalized glass coverslips (30 mm diameter) were placed onto the droplets, ensuring that the solution fully contacted the entire coverslip. After 15 minutes of polymerization, coverslips were carefully removed using a razor blade, washed with water, and dried at 45 °C for 20 to 30 minutes. Next, coverslips were placed in a 6-well plate and 20 µL of lipid solution was pipetted onto the gel. A glass Drigalski spatula was used to evenly spread the solution across the PAA gel and the solvent was evaporated in a vacuum chamber for 15 minutes. 750 µL of buffer was carefully added to a well containing the gels and incubated overnight.

**Figure 2:**
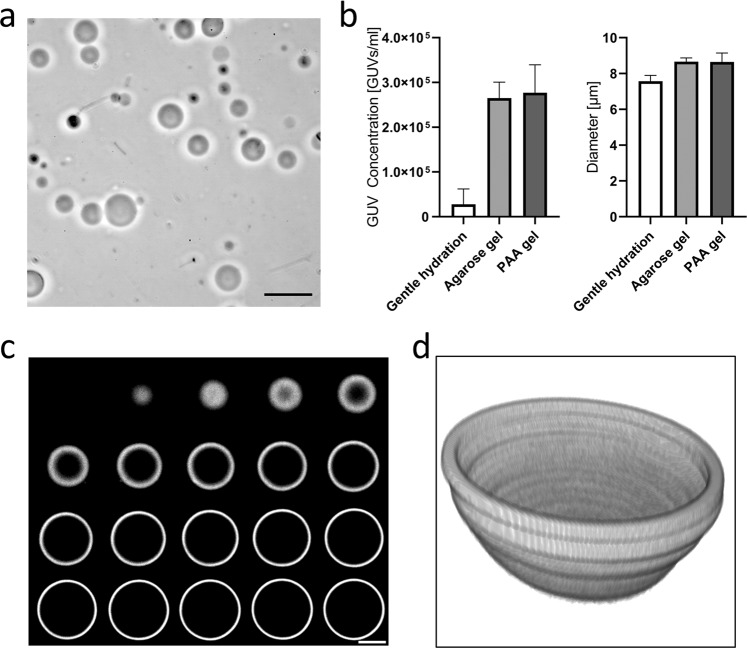
PAA hydrogel-assisted GUV generation. (**a**) Hydration of dried lipid-coated PAA hydrogels renders large populations of free-floating GUVs, as seen by phase contrast microscopy. (**b**) GUV densities are significantly larger using the agarose and PAA methods over gentle hydration on glass. Nevertheless, the mean diameter of the formed GUVs was similar, independently of the method used. (**c**) High magnification confocal imaging of the GUVs revealed normal morphologies of GUVs obtained using our method. (**d**) 3D reconstruction of the lower half of a GUV generated using PAA hydrogels. Scale bars represent 20 μm in (**a**) and 4 μm in (**c**).

Giant unilamellar vesicles (GUVs) are a model system widely used in the fields of membrane biophysics^23,24^, *in vitro* reconstitution of cellular machineries^25,26^, and drug delivery^6,8^, among others. We have developed a simple and robust method for the reproducible production of large populations of free-floating GUVs in buffers of physiological ionic strength based on the gentle hydration of lipid layers deposited on polyacrylamide (PAA) gels (Fig. 1). The method is similar to that proposed by Horger and colleagues^19^, as it is based on the rehydration of a polymeric gel onto which lipids were previously spread (Fig. 1). However, unlike agarose, PAA hydrogels are formed by the covalent reaction of its components, acrylamide (AA) and bis-acrylamide (BAA), and are therefore less likely to incorporate into the vesicles, compared to the aforementioned agarose method^20^. We compared the concentration and mean diameter of the GUVs obtained on PAA gels and using other methods for bulk GUV preparation, namely agarose hydration^19^ and gentle hydration on glass^27,28^. Experiments were carried out using DOPC lipids mixed with 0.5% fluorescently-labeled phosphoethanolamine (Rho-PE) and PBS as the hydration buffer. The quantification was done using a Coulter cell counter (ScepterTM, EMD Millipore), which estimated the concentration of cell-sized vesicles in the range of 6 to 36 μm diameter. For the PAA samples, a ratio of acrylamide (71.08 g/mol) to bisacrylamide (154.17 g/mol) of 6:0.06 was utilized (stiffness of 4 kPa and medium pore size), corresponding to formulation 5 in Table 1. The results showed larger GUV densities in the agarose and PAA hydrogel methods over gentle hydration (Fig. 2a,b). Both phase contrast and spinning disc confocal microscopy (iMic, TILL) revealed regular GUV morphologies (Fig. 2a,c,d and Supplementary Figs. 1 and 2).

**Table 1:** Polyacrylamide solutions utilized in the present study.

Sample	Acrylamide (µL)	Bis-acrylamide (µL)	Water (µL)	AA:BAA	Pore Size	Stiffness (kPa)
1	75	50	875	3: 0.1	Small	1–2
2	100	30	870	4: 0.06	Medium	1–2
3	125	15	860	5: 0.03	Large	1–2
4	100	200	700	4: 0.4	Small	4
5	150	30	820	6: 0.06	Medium	4
6	250	10	740	10: 0.02	Large	4
7	150	225	625	6: 0.45	Small	13
8	250	50	700	10: 0.1	Medium	13
9	500	15	485	20: 0.03	Large	13

Polyacrylamide (PAA) gels were prepared from acrylamide (AA, 40% w/v, Sigma A4058) and N,N-methylenebis-acrylamide (BAA, 2% w/v, Sigma M1533) stock solutions. 1 mL of the different working solutions were prepared and degassed before polymerization was initiated by the addition of 10 µL ammonium persulfate (APS, Sigma) and 1 µL N,N,N,N-tetramethylethylenediamine (TEMED, Bio-Rad 7570016). Three different pore sizes were generated for each of the 1–2, 4, and 13 kPa PAA stiffness values^29^.

### PAA pore size and stiffness have minimal impact on GUV formation

**Figure 3:**
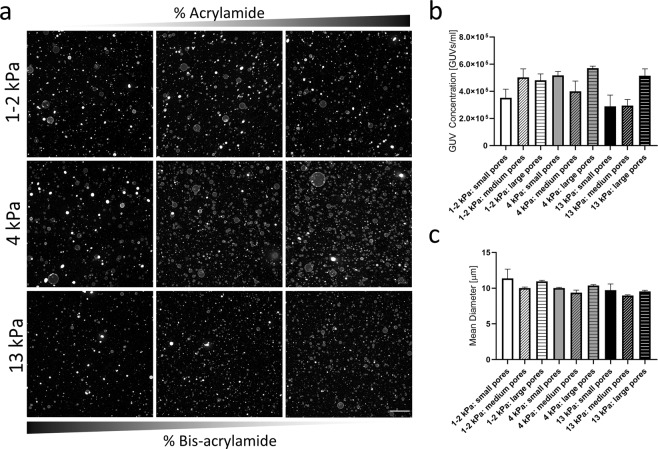
Size-quantification of GUVs generated on PAA substrates of varying stiffness and pore size. (**a**) Representative low magnification images of GUVs formed on PAA gels with different elastic moduli and pore sizes. Increasing AA:BAA ratio indicates a larger pore size. (**b**) Quantification of GUV concentration and mean diameter (**c**) using a Coulter counter reveals similar values independently of the PAA formulation used.

We next analyzed the impact of stiffness and porosity of the PAA gels on the yield and size of the generated GUVs. We used nine different PAA formulations and quantified the generated vesicles in terms of concentration and size. The stiffness values of the tested gels were 1–2 kPa, 4 kPa, and 13 kPa; each mixture was prepared using three different crosslinker ratios to generate small, medium, and large pore sizes (Table 1)^29^. Higher PAA stiffness values (30 kPa) resulted in lower GUV yields, and they were therefore omitted from this study. Experiments were carried out using DOPC lipids mixed with 0.5% Rho-PE and PBS as the hydration buffer, and the resulting GUV concentration and size distribution were assessed again using a Scepter™ system. The obtained data revealed no significant differences in concentration and size distribution of the GUVs independently of the PAA formulation used (Fig. 3a). Although large differences were observed between conditions, no clear correlations could be observed, as the GUV concentration was larger than 2.5 × 10^5^ GUVs/mL for all tested conditions (Fig. 3b). In turn, the differences in the mean diameter of the GUVs were minimal between conditions and ranged from 9.0 to 11.4 μm (Fig. 3b,c).

### PAA hydration renders predominantly unilamellar vesicles

**Figure 4:**
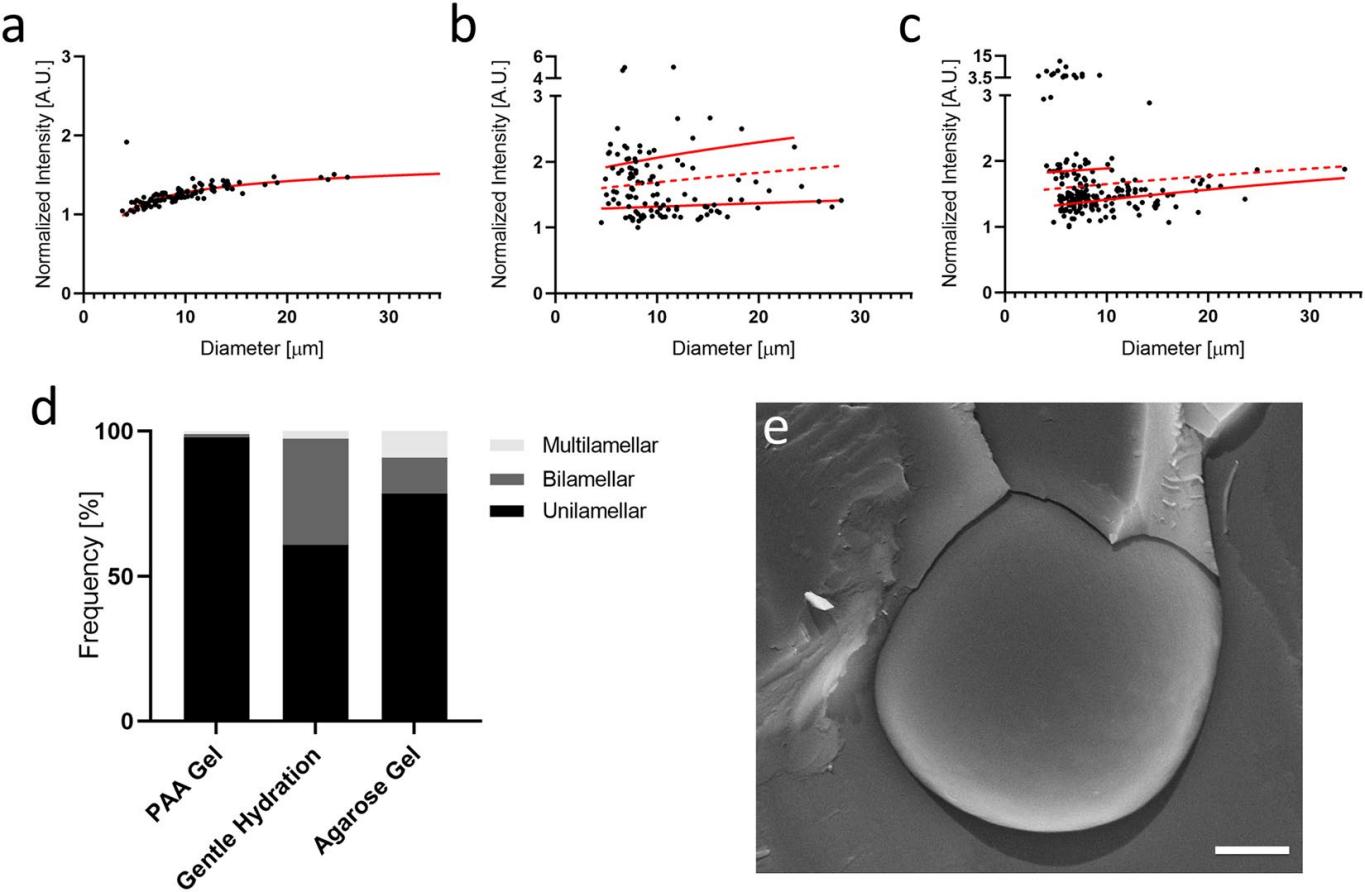
Quantitative and qualitative analysis of GUV lamellarity. The integrated membrane fluorescence signal of GUVs generated using the PAA gel (**a**), gentle hydration (**b**), and agarose gel (**c**) methods was used to determine the lamellarity of the vesicles. A logarithmic line of best fit (red lines in **a**–**c**) was applied to the unilamellar and bilamellar vesicle populations for each of these techniques, and a line halfway between each differentiates unilamellar and bilamellar vesicles (dotted red line). The quantification of unilamellar, bilamellar, and multilamellar vesicles revealed a predominantly unilamellar GUV population in case for the PAA method (**d**). (**e**) Representative cryo-SEM image of a unilamellar vesicle generated by the PAA gel technique presented here. Scale bar represents 1 μm.

For most applications using GUVs, it is important that the formed vesicles consist of a single lipid bilayer to accurately mimic a mammalian cell membrane. We therefore compared the lamellarity of the GUVs generated on PAA gels with that obtained using agarose hydration^19^ and gentle hydration on glass^27,30^. To quantify lamellarity, we used DOPC lipids doped with 0.5% Rho-PE, which were deposited on either dried PAA gels of medium pore size and stiffness of 4 kPa (formulation 5 in Table 1), 1% dried agarose gel, or directly on glass. Rehydration was done using a 100 mOsm sucrose solution and GUVs were allowed to form overnight. Images of the mid-plane region of 100 to 200 seemingly unilamellar GUVs per condition were acquired using a spinning disc confocal microscope and their membrane fluorescence was quantified using the semi-automatic ImageJ^31^ plugin “Radial profile angle” (Supplementary Fig. 3). For all three methods, vesicles with evident defects were excluded from the analysis. The fluorescence values of each vesicle were plotted against their diameter and a logarithmic line of best fit was applied to the populations^19^ (Fig. 4a–c). A line halfway between the fitting curves was applied to discriminate between unilamellar and bilamellar vesicles (dotted line in Fig. 4a–c). For all three methods tested, there is a logarithmic increase of relative fluorescent intensity, which agrees with the literature^32^. Interestingly, no significant multilamellar population was detected for the PAA gel method (Fig. 4a,d), while the gentle hydration and agarose gel methods created significant amounts of bilamellar and multilamellar vesicles (Fig. 4b–d). Cryo-SEM (Scanning electron microscopy) imaging of the GUVs generated using PAA gels further confirmed their primarily unilamellar nature (Fig. 4e).

### PAA hydration results in large GUV populations independently of the lipid and buffer types used

**Figure 5:**
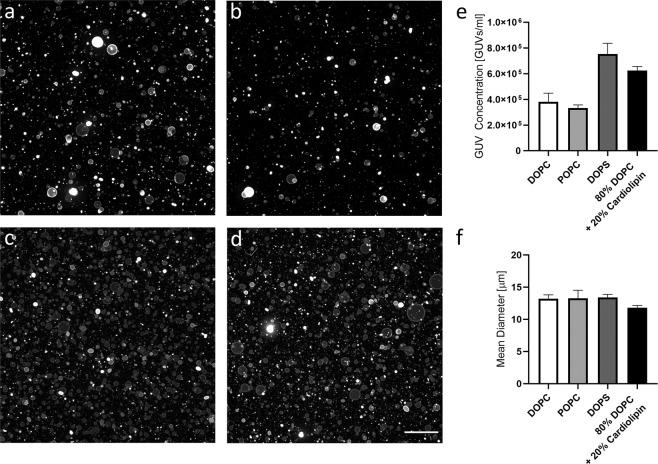
Formation of GUVs composed of different lipid mixtures. Representative low magnification confocal images of GUVs formed using DOPC (**a**), POPC (**b**), DOPS (**c**), and 80% DOPC + 20% cardiolipin (**d**). (**e**) Quantification of GUV concentration and mean diameter (**f**) using a Coulter counter. Scale bar represents 100 μm.

We further tested the versatility of the PAA method towards the generation of large GUV populations composed of different lipid types. We found that high quantities of GUVs were obtained using a wide range of lipid types including neutral lipids (DOPC and POPC), negatively charged lipids (DOPS), and the commonly utilized combination of DOPC with cardiolipin (80% DOPC + 20% cardiolipin)^33–35^ (Fig. 5). All lipid mixtures tested resulted in GUV concentrations above 3 × 10^5^ vesicles/mL with mean diameters between 11.8 and 13.4 μm (DOPC/Cardiolipin and DOPS, respectively).

**Figure 6:**
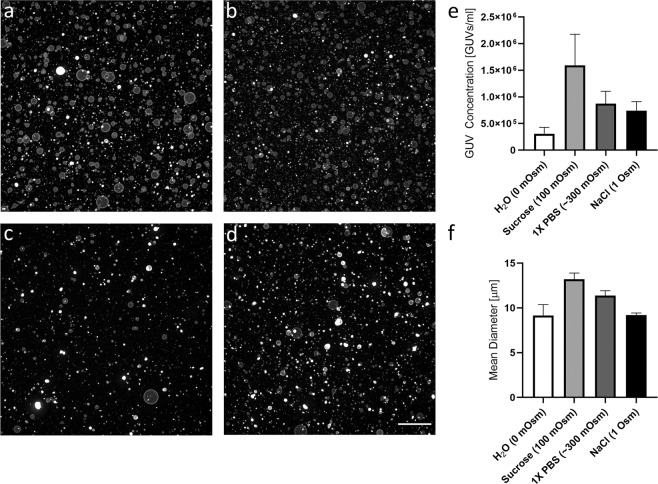
Formation of GUVs using buffers with different ionic strengths. Representative low magnification confocal images of GUVs formed using (**a**) distilled water (0 mOsm), (**b**) 100 mM sucrose (100 mOsm), (**c**) PBS (300 mOsm), and (**d**) 0.5 M NaCl (1 Osm). (**e**) Quantification of GUV concentration and mean diameter (**f**) using a Coulter counter. Scale bar represents 100 μm.

To validate the robustness of the method towards different buffer compositions, we tested the impact of rehydration solutions with osmolarity values ranging from zero (0 mOsm, distilled water) to 1000 mOsm (0.5 M NaCl) on the generation of DOPC GUVs (Fig. 6a–d and Supplementary Fig. 2). We quantified the concentration and the diameter using a Scepter^™^ device and found that distilled water consistently rendered the lowest GUV concentrations and mean diameters, while the highest values were obtained with 100 mOsm sucrose. A buffer of physiological ionic strength, represented by PBS (300 mOsm), performed the second best with respect to GUV concentration (8.7 × 10^5^ vesicles/mL) and resulted in GUVs with mean diameters of 11.4 μm. Even as ionic strength exceeded physiological levels (1 Osm NaCl), GUV formation was not hindered, and significant amounts of GUVs with a mean diameter of approximately 9.2 μm were generated (black bars in Fig. 6e,f).

## Discussion

In this study, we have established a simple method for the reliable production of free-floating giant unilamellar vesicles (GUVs) under conditions of physiological ionic strength. The technique utilizes soft polyacrylamide (PAA) gels ranging in stiffness between 1 and 13 kPa, and varying porosity based on cross-linking density. Upon PAA polymerization, the gels are dried, lipids are evenly spread on the surface, and a buffer of choice is used to rehydrate the lipid film. This technique renders large numbers of GUVs (>10^5^ GUVs/mL) for a wide range of lipid types, including charged lipid types, using buffers of physiological ionic strength. We have confirmed that GUVs are generated for a range of gel stiffness values (1–13 kPa) and porosities with a minimal impact on GUV diameter or concentration. Importantly, we have further demonstrated that the GUVs produced with the PAA method show a higher percentage of unilamellar vesicles when compared to current standards in the field.

Until recently, the generation of GUVs in buffers of physiological ionic strength has proven to be a challenge, especially for methods such as gentle hydration and electroformation^12,13^. Nevertheless, the production of GUVs under physiological conditions is crucial for artificial cell studies, as low ionic strength can hinder protein stability^12,36^, vary membrane properties^12^, and complicate protein polymerization^37^. Since Reeves and Dowben first reported a method to generate GUVs by the controlled hydration of a thin dry film of egg yolk phosphatidylcholine (egg PC) deposited on a glass surface^27^, several adaptations of the gentle hydration technique have been described^30,38^. While this technique is simple and broadly applicable, it is not compatible with many charged lipids when rehydration buffers of physiological strength are utilized^30^. Recently, electroformation methods have successfully generated significant GUV populations both from charged lipid types^16,17^ and in physiologically relevant buffers^13^; however, only few studies incorporate both of these parameters into the GUV formation process. As an alternative method for the production of GUVs considering these two criteria, the polyacrylamide method described here yields significant GUV formation for buffers of physiological ionic strength, and even up to approximately three times physiological ionic strength. Additionally, charged lipids do not seem to interfere with the vesicle formation process; in fact, negatively charged lipids (DOPS) consistently generated the largest number of GUVs out of all tested lipid combinations.

In addition to vesicle generation in buffers of physiological ionic strength, the ability to form homogeneous populations of unilamellar vesicles is an important consideration for recapitulating cellular systems. This is especially important in studies investigating biophysical aspects, as lamellarity alters the mechanical properties and forces required for vesicle deformation^24^, and can interfere with applications such as actin polymerization and the study of protein dynamics^39^. Due to the difficulty in controlling lamellarity, hydration methods often require additional processing methods, such as extrusion^8,10^, to obtain unilamellar populations. In our hands, using PAA gels for hydration produces a uniform population of unilamellar GUVs, whereas the method using agarose gels^19^ and a representative gentle hydration technique both showed significant populations of bi- and multi- lamellar vesicles.

One drawback of the agarose gel model is the incorporation of part of the hydrogel constituents into the formed GUVs. This phenomenon was revealed by Lira *et al*.^20^, who showed that agarose incorporation led to mechanically heterogeneous vesicle populations. Since then, a number of alternative substrates have been proposed (e.g. poly(vinyl alcohol) or dextran(ethylene glycol)), which overcame this problem of the original technique^21,22^. Currently, the mechanical homogeneity of the GUVs produced with our polyacrylamide method has not been rigorously assessed. Likewise, the chemical purity of the GUVs and the extent of PAA incorporation have not been quantified, but the covalent reaction of the PAA hydrogel components reduces the likelihood of this occurring, compared to the aforementioned agarose method^19^. Additionally, the complexity and requirement of uncommon chemicals of some of these new approaches have hindered their broad adoption in the field. As with hydration of agarose films, the use of soft polyacrylamide hydrogels to promote GUV formation presented here only employs common chemical reagents in most wet laboratories. Although microfluidic approaches offer more precise control for the generation of predominantly unilamellar vesicles^40,41^, production of asymmetric vesicles^7^, or successful macromolecule encapsulation^40,41^, these fabrication procedures can be laborious and costly, and require technical expertise.

In their work, Horger *et al*. further compared the use of PAA to agarose gels for GUV formation^19^, and reported a lower yield for the PAA hydrogels generated using an AA:BAA ratio of 37.5:1. Using a range of PAA hydrogel stiffness and pore size values (shown in Table 1) we were able to generate significant GUV populations with minimal variation between conditions. However, in our hands the 37.5:1 PAA hydrogels did neither render large populations of GUVs, probably due to the increased stiffness of this gels. In fact, the use of gels with stiffness values higher than 30 kPa dramatically reduced the generation of GUVs. Another key difference between our PAA method and the PAA method proposed by Horger *et al*. is that the latter only partially dried the PAA gels before depositing a lipid layer on top^19^. To prevent gel detachment, we used glutaraldehyde-functionalized coverslips, thus allowing for complete drying of the PAA gel. By doing so, we were able to spread the lipids, suspended in a chloroform and methanol solution, on a fully dried gel and therefore promote significant GUV generation.

In summary, our GUV technique provides a reproducible method for the generation of large cell-sized populations of largely unilamellar vesicles for a broad range of lipid types and buffers. The PAA method can be easily employed with standard laboratory reagents, and can serve as a versatile platform for many applications of GUVs and model cell systems, such as artificial machinery, actin dynamics, and membrane biophysics.

## Methods

### Lipids

The lipids (Avanti Polar Lipids Inc.) used in this study were: 1,2-dioleoyl-sn-glycero-3-phosphocholine (DOPC), 1-palmitoyl-2-oleoyl-glycero-3-phosphocholine (POPC), 1,2-dioleoyl-sn-glycero-3-phospho-L-serine (DOPS), and 1′,3′-bis[1,2-dimyristoyl-sn-glycero-3-phospho]-glycerol (cardiolipin). Mixtures used for imaging included 0.5% of the auto-fluorescent lipid 1,2-dipalmitoyl-sn-glycero-3-phosphoethanolamine-N-(lissamine rhodamine B sulfonyl) (Rho-PE) to facilitate the observation of the GUVs under a spinning disc confocal microscope (iMic, TILL Photonics).

### Polyacrylamide preparation on glass coverslips

Circular glass coverslips with a diameter of 30 mm (Menzel Gläser #1) were chemically modified to allow for covalent binding of polyacrylamide substrates using previously described protocols^42,43^. Briefly, coverslips were rinsed with ethanol and distilled water and placed in an oxygen plasma oven (Diener Elektronic) for 5 minutes. Activated coverslips were incubated at room temperature in a 10% APTES ((3-Aminopropyl)trimethoxysilane, Sigma A3648) solution in ethanol for 15 to 30 minutes, washed with ethanol and distilled water, and further incubated for 15 to 30 minutes in a 2.5% glutaraldehyde (GA, Sigma G6257) solution in PBS. Finally, the coverslips were washed in distilled water, dried, and stored at 4 °C.

### Agarose hydrogel preparation on glass coverslips

Agarose gels were prepared following the protocol previously described by Horger *et al*. in 2009^19^. Briefly, 30 mm diameter coverslips were cleaned with ethanol and distilled water and activated in the oxygen plasma oven for 1 min. 300 μL of a pre-heated solution of 1% ultra low gelling agarose (Sigma A2576) was deposited on the slip and carefully spread out using a glass Drigalski spatula. The coverslips with the agarose solution facing upwards were placed on a hotplate at 50 °C for 1 hour. This process was repeated 4 more times for a total of 5 layers of agarose gel. Giant vesicles were generated using the same approach as with the PAA gels.

### GUV generation

Lipids were dissolved in a 10:1 chloroform/methanol mixture to a concentration of 1.5 mM and evenly spread on the different hydrogels with a glass Drigalski spatula. The solvent was evaporated by incubation of the gels for 15 minutes in a vacuum chamber. 750 µL of buffer was added to each well containing the gels. GUVs were allowed to form overnight although significant vesicle formation could be observed after shorter times (less than 2 hours).

### Confocal imaging

50 μL of the GUV suspension (containing 0.5% rhodamine labeled lipids) was deposited on a 25 mm glass coverslip in a sample holder (SKE Advanced Therapies) and an 18 mm coverslip was placed on top. The coverslips were previously incubated in a 1% BSA aqueous solution for 1 hour to prevent non-specific binding of GUVs to the glass surface. A spinning disc confocal microscope (iMic, TILL Photonics) equipped with a 10x air objective (N.A. 0.4) and 60x water immersion objective (N.A. 1.2) was used to capture images and image stacks of the GUV suspensions. A maximum intensity projection was used to obtain representative images at 10x magnification. Confocal stacks acquired using a 60× (N.A. 1.35) objective were used to asses GUV morphology and generate 3D reconstructions.

### Phase contrast imaging

GUVs were formed using DOPC lipids spread on hydrogels composed of an AA:BAA ratio of 6:0.06 (formulation 5 in Table 1), and rehydrated using a 100 mOsm sucrose solution. After overnight formation, the GUV suspension was mixed with an equal amount of 100 mOsm PBS, centrifuged at 500 g for 5 minutes, and resuspended in either 100 mOsm PBS or 100 mOsm glucose to facilitate visualization using phase contrast microscopy. GUVs were imaged using a TS100 microscope (Nikon).

### Lamellarity quantification

Lamellarity experiments were carried out using DOPC lipids with 0.5% rhodamine labeled lipids, obtained using the here described PAA method (formulation 5 in Table 1), the gentle hydration of a lipid film spread on glass (referred to as ‘gentle hydration method’ in our manuscript)^44^, and the agarose gel method^19^. In all cases, lipid films were rehydrated with 100 mOsm sucrose, chosen for its moderate ionic strength, as an intermediate value between water and physiological ionic strength. For lamellarity quantification of GUVs, a single image was captured at the mid-plane of a number of seemingly unilamellar GUVs. We then adapted the methods described by Chiba *et al*.^32^ and Ewins *et al*.^45^ using the semi-automatic ImageJ plugin “Radial Profile Angle”^31^. Briefly, once a circular outline was chosen for each GUV, the algorithm integrated the signal around the complete circumference of the circle and reported an integrated intensity for the given radius. This was repeated for every pixel in the radial direction, until the initial specified radius as achieved. The plugin subsequently provided the normalized integrated intensity as a function of the radius (Supplementary Fig. 3). For each of the 3 methods, the intensity values were normalized with respect to the lowest intensity point and plotted against vesicle diameter. A logarithmic line of best fit was applied to the unilamellar and bilamellar vesicle populations for each of these techniques. Subsequently, a line halfway between each of these two fits was applied to better differentiate unilamellar and bilamellar vesicles (indicated by a dotted red line in Fig. 4)^32^. If a data point fell above the dotted line, it was classified as bilamellar and below the dotted line was classified as unilamellar.

### Lipid and buffer type experiments

PAA substrates with an elastic modulus of 1–2 kPa and small pore size were employed for lipid type and ionic strength experiments. To assess the effect of lipid type on GUV formation, GUVs were generated with neutral lipids (DOPC and POPC), negatively charged lipids (DOPS), and the commonly utilized combination of DOPC with cardiolipin (80% DOPC +20% cardiolipin)^33–35^. Lipid films were rehydrated with 750 µL of PBS. For assessment of ionic strength of the rehydrating solution, buffers ranging from 0 mOsm to 1 Osm were utilized.

### Cryo-SEM

For cryo-SEM, GUVs were prepared in 150 mM sucrose dissolved in PBS to reduce the formation of ice crystals upon freezing. GUV suspensions were pipetted into the 100 µm deep slit of two aluminium specimen slit carriers (Engineering Office M. Wohlwend GmbH, Sennwald, Switzerland). The carriers then were assembled by facing the slits towards each other. Short filter tip pieces (about 1 mm in length) were placed into the alignment holes of the slit carriers and wetted with buffer. The assembled sandwich was subsequently transferred into the hole of a 3 mm middle plate, a 100 µm stainless still spacer ring was added on top, and the sample was frozen using a HPM 100 high-pressure freezer (Leica Microsystems, Vienna, Austria) without ethanol as synchronization fluid. For freeze-fracturing, frozen sample sandwiches were attached to a custom-made vice holder in liquid nitrogen and transferred to a BAF 060 freeze-fracturing device using the VCT 100 cryo transfer system (Leica Microsystems). Samples were fractured at −120 °C and immediately coated by electron beam evaporation with 2.5 nm of platinum/carbon at an angle of 45° followed by 5 nm carbon at an angle of 45° and stage rotation of 40 rpm. Subsequently, samples were transferred onto the cryo-stage of a Zeiss Auriga 40 CrossBeam SEM (Zeiss, Oberkochen, Germany) and imaged at −115 °C and at an acceleration voltage of 5 kV using the Inlens secondary electron detector.

### Size distribution analysis

A Scepter™ 2.0 Handheld Cell Counter (EMD Millipore) was used to quantify the mean diameter and concentration of the GUVs. This device, traditionally used in mammalian cell culture, is based on the Coulter Principle; as each cell passes through an aperture, a voltage spike is recorded and its magnitude is correlated to particle size^46,47^. 60 µm tips were used, which are capable of detecting particles between 6 and 36 µm. Upon data collection, the sample was gated to include the region between 6.9 to 36 µm, as there was frequently significant noise in the 6 to 6.9 µm interval. Each experimental condition was performed in triplicate and three Scepter™ readings were performed for each sample. The standard deviations of the GUV concentration and reported mean diameter value were calculated for the nine readings in each experimental group.

## Supplementary information

Supplementary information.

Supplementary information 2.

Supplementary information 3.

Supplementary information 4.

Supplementary information 5.

## Data Availability

All data generated during this study are available from the corresponding author, U.S., upon reasonable request.
