# Supplementary material for: Facile generation of giant unilamellar vesicles using polyacrylamide gels
Source: Sci Rep. 2020 Mar 16;10:4824. doi: 10.1038/s41598-020-61655-2 (PMC7075891; doi:10.1038/s41598-020-61655-2)
Supplement: Supplementary file 1 — Supplementary information. [file 41598_2020_61655_MOESM1_ESM.pdf]

Title

Facile generation of giant unilamellar vesicles using polyacrylamide gels

Authors

Eric Parigoris, Daniel L Dunkelmann, Allan Murphy, Nino Wili, Andres Kaech, Claudia Dumrese, Noemi Jimenez-Rojo, Unai Silvan.

Supplementary Information

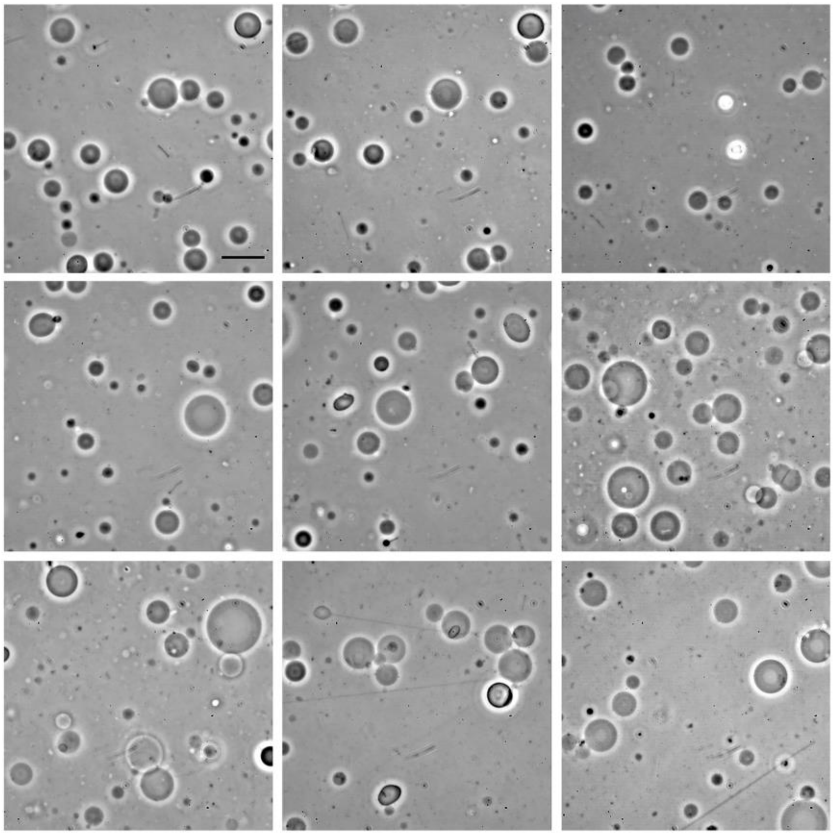

Supplementary Figure 1. – Phase contrast images of GUVs. Image gallery of GUVs generated using the PAA method visualized under a phase contrast microscope. To facilitate phase contrast visualization, GUVs were filled with 100 mOsm sucrose and resuspended in either 100 mOsm PBS (panels 1-5) or 100 mOsm glucose (panels 6-9). Scale bar represents 20 µm.

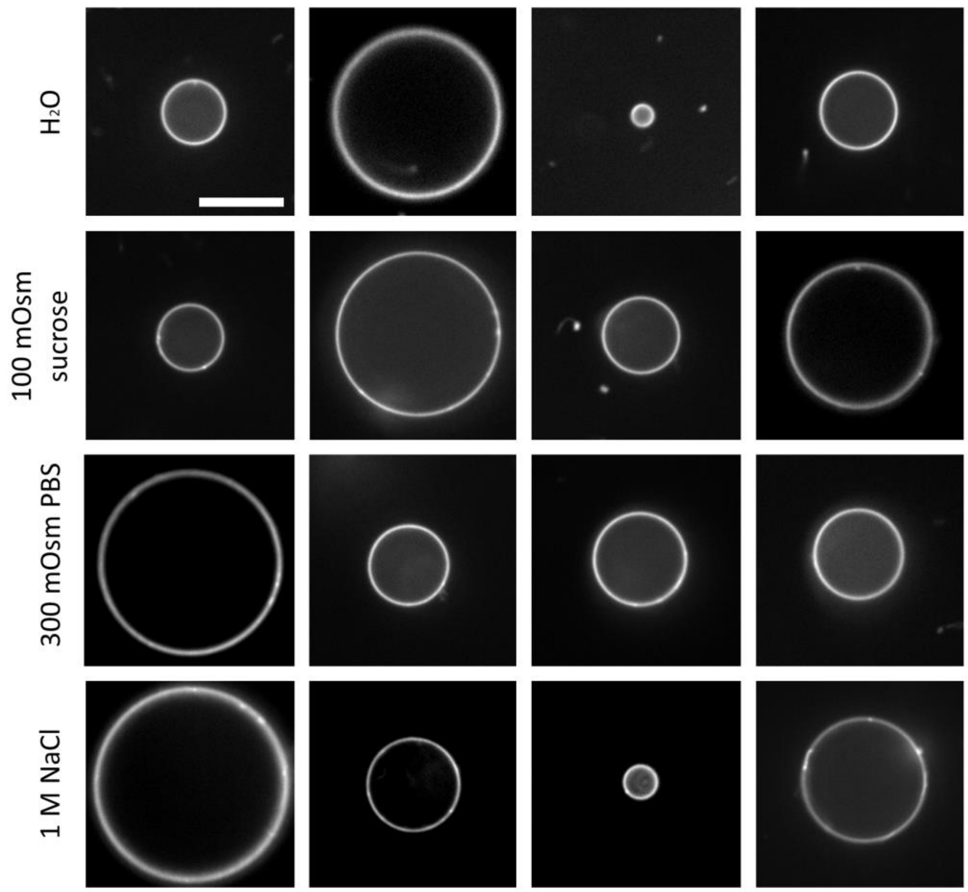

Supplementary Figure 2. – Confocal images of GUVs. Confocal images of the mid-plane of GUVs generated with the PAA method. Scale bar represents 10 µm.

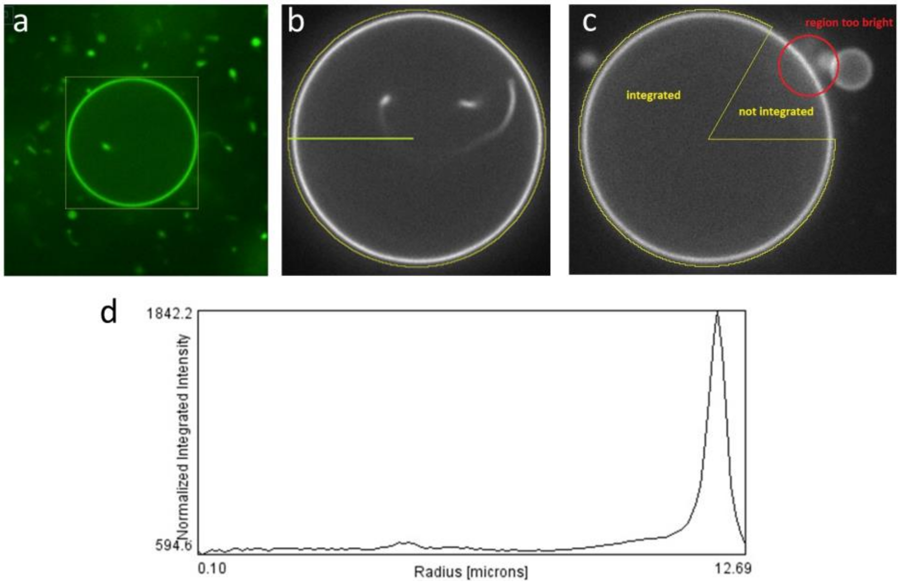

Supplementary Figure 3. – Lamellarity quantification with Image J. (A) Selection of square region of interest around the GUV. (B) Selection of circumference of GUV for intensity integration. (C) Exclusion of a region for intensity integration due to small vesicle fusion or signal interference from a nearby GUV. (D) Plot of normalized integrated intensity versus radius. The peak value was chosen to represent the membrane intensity.
