# Supplementary figures and images for: Facile generation of giant unilamellar vesicles using polyacrylamide gels
Source: Sci Rep. 2020 Mar 16;10:4824. doi: 10.1038/s41598-020-61655-2 (PMC7075891; doi:10.1038/s41598-020-61655-2)

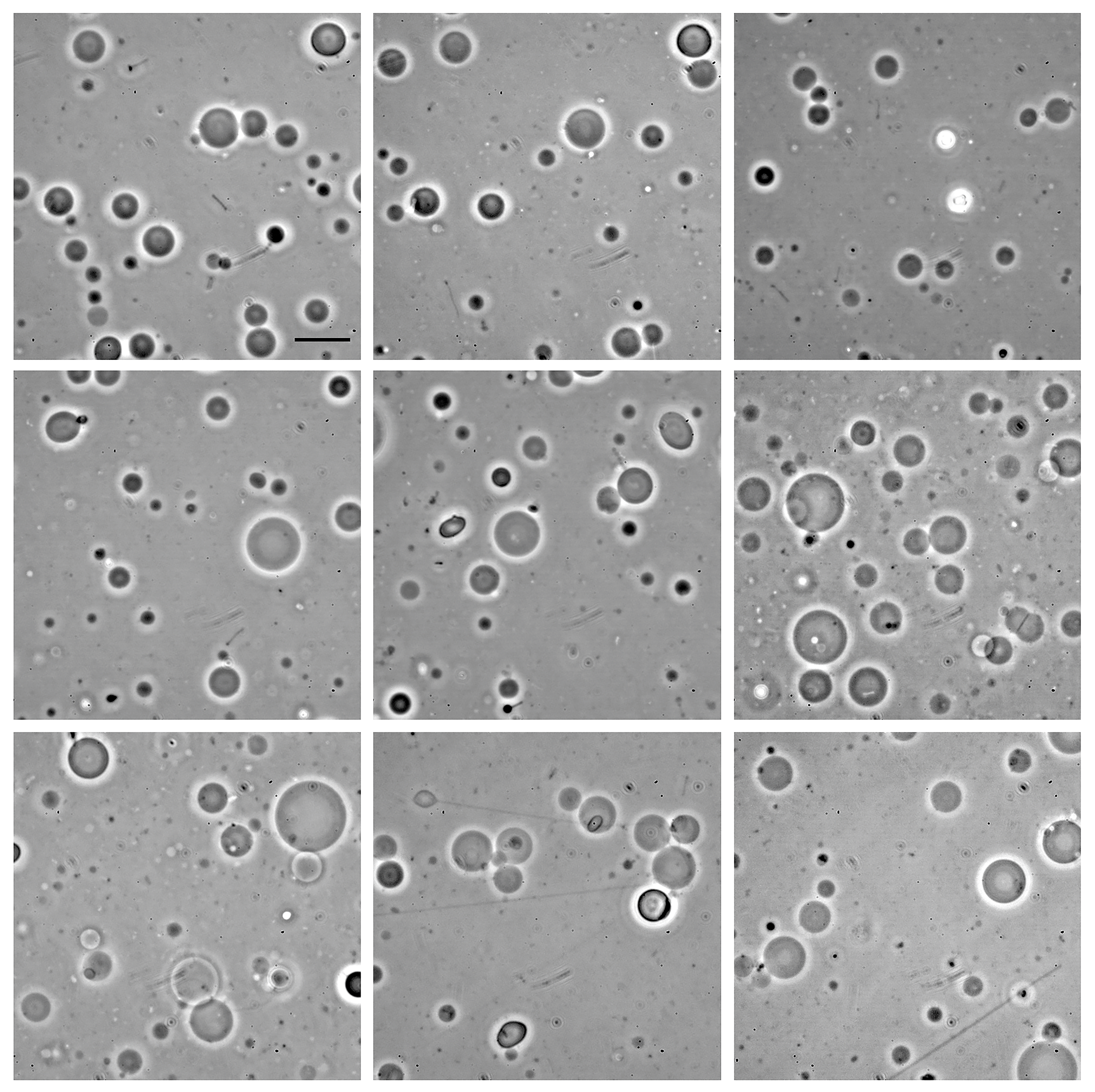

Supplement: Supplementary file 2 — Supplementary information 2. [file 41598_2020_61655_MOESM2_ESM.tif]

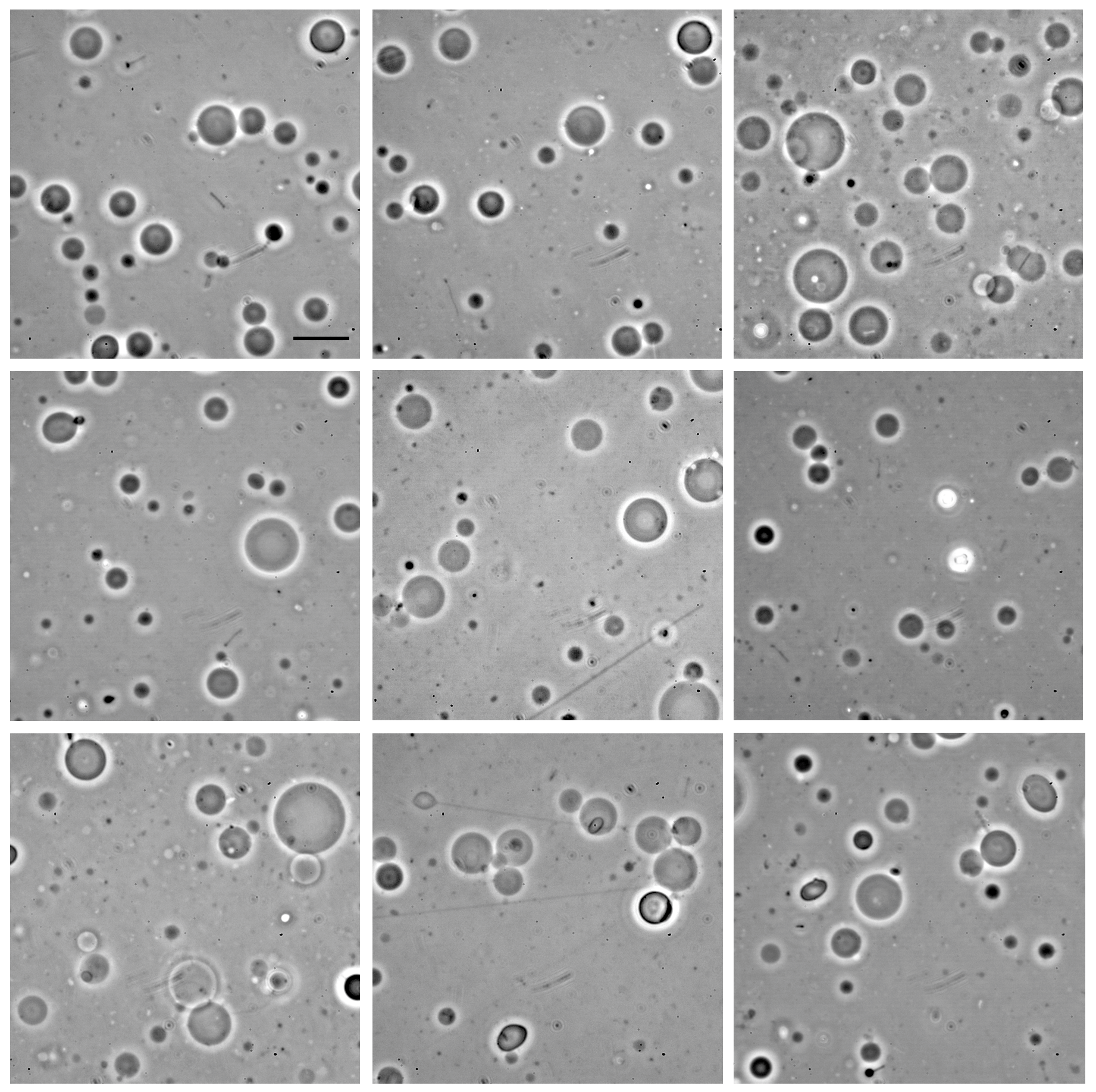

Supplement: Supplementary file 3 — Supplementary information 3. [file 41598_2020_61655_MOESM3_ESM.tif]

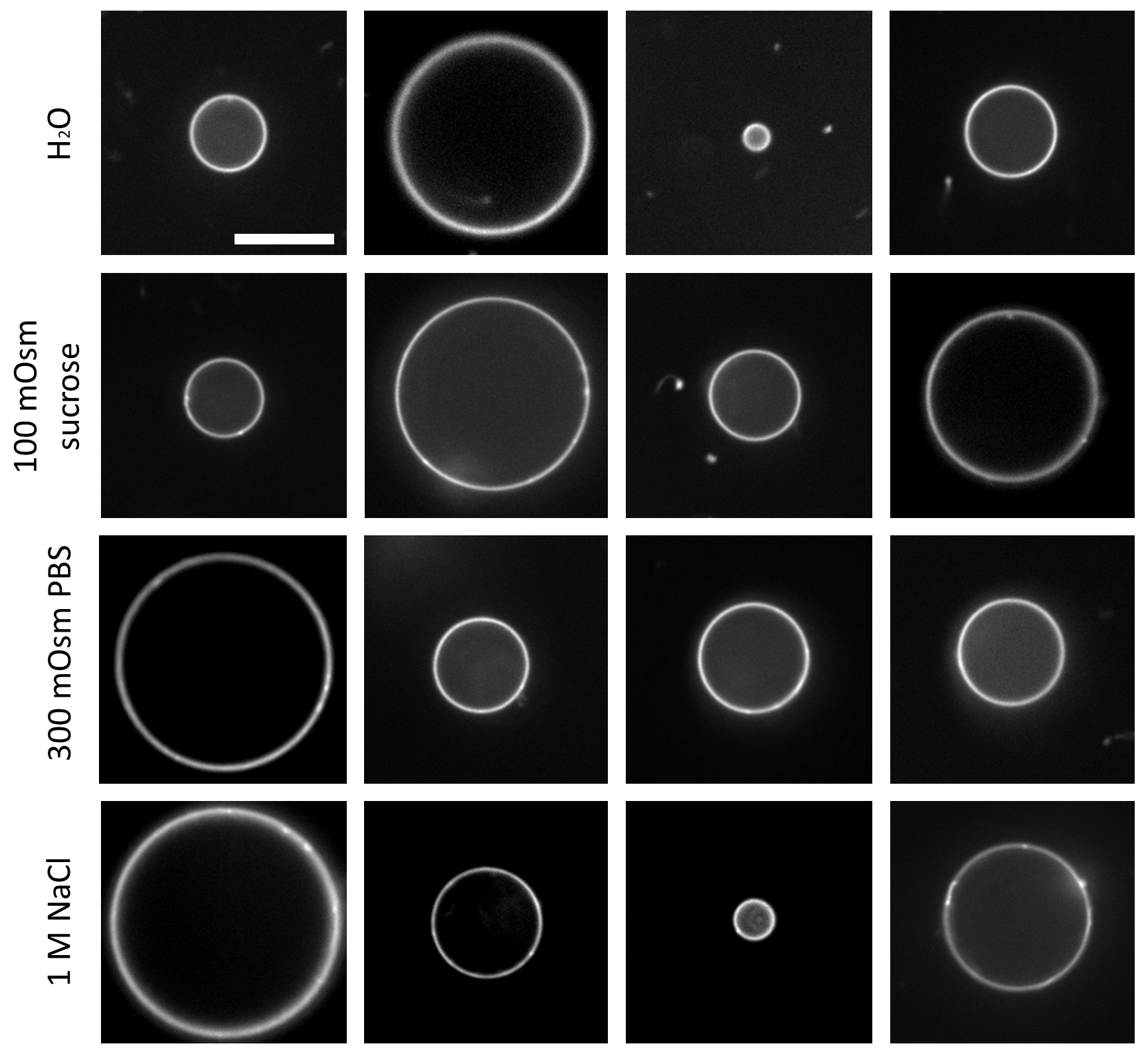

Supplement: Supplementary file 4 — Supplementary information 4. [file 41598_2020_61655_MOESM4_ESM.tif]

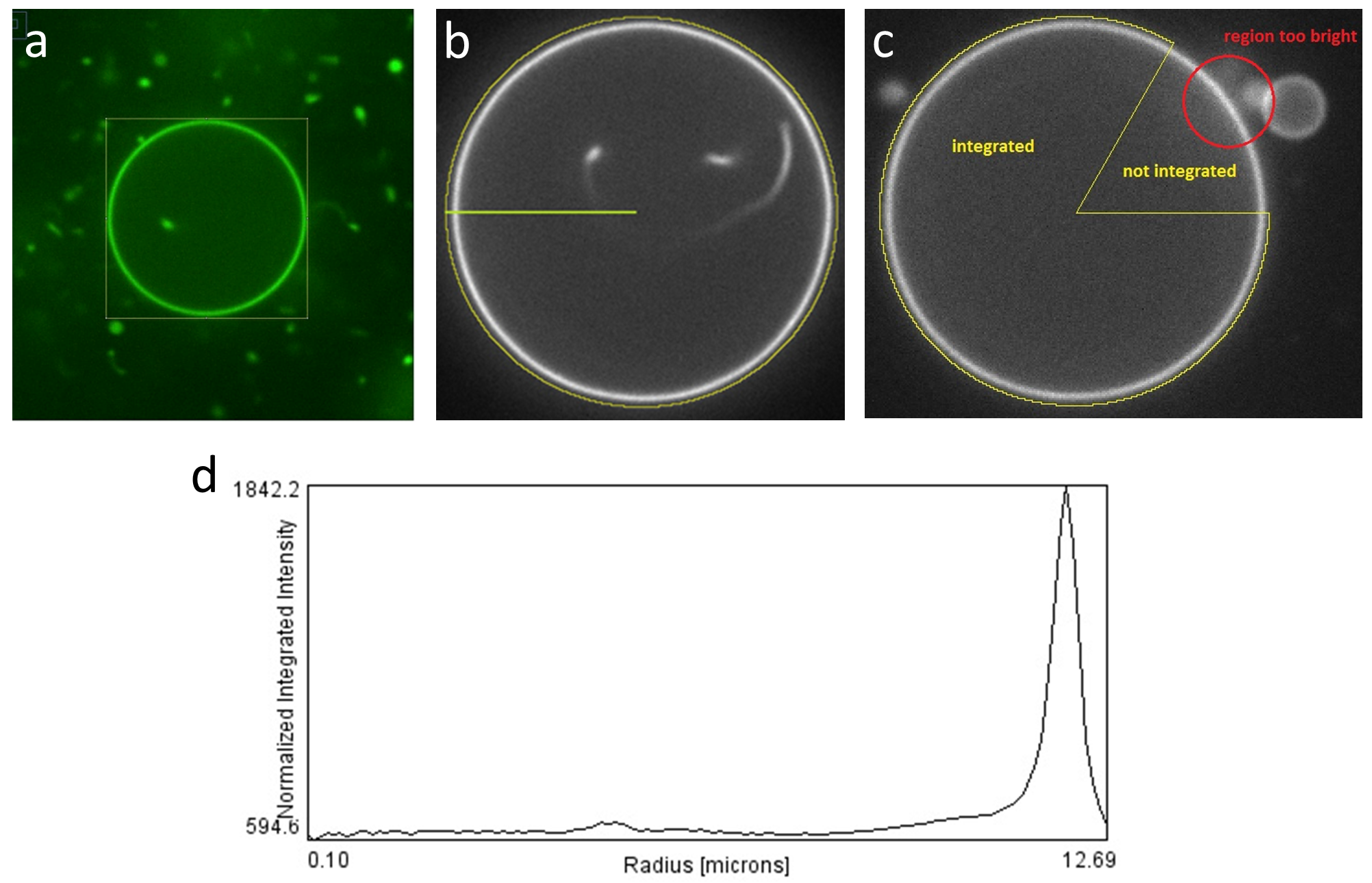

Supplement: Supplementary file 5 — Supplementary information 5. [file 41598_2020_61655_MOESM5_ESM.tif]
